# Regenerative potential of human dental pulp stem cells in the treatment of stress urinary incontinence: In vitro and in vivo study

**DOI:** 10.1111/cpr.12675

**Published:** 2019-09-25

**Authors:** Alessio Zordani, Alessandra Pisciotta, Laura Bertoni, Giulia Bertani, Antonio Vallarola, Daniela Giuliani, Stefano Puliatti, Daniela Mecugni, Giampaolo Bianchi, Anto de Pol, Gianluca Carnevale

**Affiliations:** ^1^ Unit of Urology ULSS1 Dolomiti Feltre Belluno Italy; ^2^ Histology Section, Department of Surgery, Medicine, Dentistry and Morphological Sciences, with Interest in Transplants, Oncology and Regenerative Medicine University of Modena and Reggio Emilia Modena Italy; ^3^ Department of Life Sciences University of Modena and Reggio Emilia Modena Italy; ^4^ Department of Biomedical, Metabolic and Neural Sciences University of Modena and Reggio Emilia Modena Italy; ^5^ Urology Unit Department of Surgery, Medicine, Dentistry and Morphological Sciences, with Interest in Transplants, Oncology and Regenerative Medicine University of Modena and Reggio Emilia Modena Italy; ^6^ Azienda USL–Institute and Health care (IRCCS) di Reggio Emilia Reggio Emilia Italy

## Abstract

**Objectives:**

To evaluate the regenerative potential of human dental pulp stem cells (hDPSCs) in an animal model of stress urinary incontinence (SUI). SUI, an involuntary leakage of urine, is due to physical stress involving an increase in bladder pressure and a damage of external urethral sphincter affecting muscles and nerves. Conventional therapies can only relieve the symptoms. Human DPSCs are characterized by peculiar stemness and immunomodulatory properties and might provide an alternative tool for SUI therapy.

**Materials and methods:**

In vitro phase: hDPSCs were induced towards the myogenic commitment following a 24 hours pre‐conditioning with 5‐aza‐2′‐deoxycytidine (5‐Aza), then differentiation was evaluated. In vivo phase: pudendal nerve was transected in female rats to induce stress urinary incontinence; then, pre‐differentiated hDPSCs were injected in the striated urethral sphincter. Four weeks later, urethral sphincter regeneration was assayed through histological, functional and immunohistochemical analyses.

**Results:**

Human DPSCs were able to commit towards myogenic lineage in vitro and, four weeks after cell injection, hDPSCs engrafted in the external urethral sphincter whose thickness was almost recovered, committed towards myogenic lineage in vivo*,* promoted vascularization and an appreciable recovery of the continence. Moreover, hDPSCs were detected within the nerve, suggesting their participation in repair of transected nerve.

**Conclusions:**

These promising data and further investigations on immunomodulatory abilities of hDPSCs would allow to make them a potential tool for alternative therapies of SUI.

## 1 INTRODUCTION

Urinary incontinence (UI), defined as the involuntary leakage of urine, affects 200 million people worldwide, with prevalence increasing with age, increases rate of skin infection and has significant psychosocial and economic burdens, leading to significant quality of life issues.

Urinary incontinence arises from dysfunctions affecting the pelvic floor, whose physiology is maintained by a fine balance among bone, muscle and nerve tissues.

The three most common types of UI are stress urinary incontinence (SUI), urge urinary incontinence (UUI) or a combination of both, that is mixed urinary incontinence (MUI). Although SUI is the most prevalent type in women,^1^ the symptoms of stress incontinence are associated with urethral hypermobility and intrinsic sphincter deficiency. While the former is caused by a weakness in the pelvic floor musculature, pelvic fascia and pubourethral ligaments, the latter is caused by pudendal nerve damage and damage to the intrinsic and extrinsic urethral sphincter, as occurring in men after radical prostatectomy.^2^ These two injuries affecting striated muscle and nerve fibres are strongly correlated with the SUI.^3^ For the prevention of male stress incontinence resulting from radical prostatectomy, some researchers have proposed intraoperative reconstructive techniques such as the posterior musculofascial reconstruction^4^ or the pubo‐prostatic ligament reconstruction.^5^

However, surgical approaches, such as tension‐free vaginal tape, transobturator slings or pubovaginal slings, remain the gold standards for SUI treatment. Most of these strategies apply local compression to the urethra in order to improve continence but do not correct the underlying muscle and nerve pathology. However, not all patients can benefit from the surgery because the procedure‐related risks (associated with the patients’ clinical comorbidities) may outweigh the benefits of this therapy.^6^ The efficacy of pharmacotherapy for SUI has been disappointing. Attempts to avoid invasive surgery and morbidity have included the use of various injectable bulking agents, including polytetrafluoroethylene, bovine collagen, silicone particles, carbon beads and autologous fat or chondrocytes.^7^ However, these treatments are performed using non‐absorbable foreign materials, and implantation of foreign materials is associated with a number of possible side effects, including infection and erosion.^8^, ^9^

Regenerative medicine may provide an exciting means to fill the therapeutic gap by restoring and maintaining normal function via direct or indirect effects on injured tissues.

So far different stem cells sources, such as muscle‐derived stem cells, adipose stem cells, bone marrow stem cells and amniotic fluid stem cells,^10^ have been evaluated in different preclinical and clinical studies demonstrating their potential to restore the urinary continence.^11^, ^12^, ^13^, ^14^, ^15^, ^16^, ^17^, ^18^, ^19^, ^20^, ^21^, ^22^, ^23^ Although these stem cells might be promising for their regenerative potential, the evaluation of further stem cells sources is under investigation. Particularly, dental pulp stem cells can be obtained through non‐invasive procedures, exert immunomodulatory properties and are able to differentiate towards different lineages. Previous findings demonstrated that hDPSCs are able to participate to the regeneration of skeletal muscle in Duchenne Muscular Dystrophy (DMD) and peripheral nerve injury animal models after reaching the commitment towards myoblasts and Schwann cells, respectively.^24^, ^25^ Even though hDPSCs were investigated in the regeneration of bladder smooth muscle, less is known about their application in animal model of SUI. To this regard, the aim of our study was to investigate whether hDPSCs are able to restore the urethral sphincter to normal histology and function in female rats after inducing SUI through pudendal nerve resection.

## 2 MATERIALS AND METHODS

### 2.1 Isolation and immune selection and stemness properties of hDPSCs

The study was approved by the Comitato Etico Provinciale—Azienda Ospedaliero‐Universitaria di Modena (Modena, Italy), which provided the approval of the protocol (ref. number 3299/CE).

Human DPSCs were isolated from third molars of adult subjects (n = 3; 18‐25 years). All subjects gave written informed consent in accordance with the Declaration of Helsinki. Dental pulp was harvested from the teeth and underwent enzymatic digestion by using a digestive solution, consisting in 3 mg/mL type I collagenase plus 4 mg/mL dispase in α‐MEM. Pulp was then filtered onto 100 μm Falcon Cell Strainers, in order to obtain a cell suspension. Cell suspension was then plated in 25 cm^2^ culture flasks and expanded in culture medium [α‐MEM supplemented with 10% foetal bovine serum (FBS), 2 mmol/L l‐glutamine, 100 U/mL penicillin, 100 μg/mL streptomycin] at 37°C and 5% CO2. Non‐adherent cells were discarded and hDPSCs were expanded upon 80% confluency. Following cell expansion, human DPSCs underwent magnetic cell sorting through MACS® separation kit. Particularly, hDPSCs were selected for the expression of STRO‐1 and c‐Kit surface antigens by using mouse IgM anti‐STRO‐1 and rabbit IgG anti‐c‐Kit primary antibodies (Santa Cruz), respectively. The following magnetically labelled secondary antibodies were used: anti‐mouse IgM, anti‐rabbit IgG (Miltenyi Biotec). hDPSCs were sequentially immune‐selected as previously described by Pisciotta et al.^26^ The expression of HLA‐ABC, HLA‐DP‐DQ‐DR and FasL expression was evaluated through immunofluorescence analyses by direct staining with mouse anti‐human HLA‐ABC, mouse anti‐human HLA‐DP‐DQ‐DR (BD Biosciences) and rabbit anti‐human FasL (Santacruz, Santa Cruz Biotechnology, Inc) antibodies.

### 2.2 Osteogenic and adipogenic differentiation

The mesenchymal ability was evaluated by inducing hDPSCs towards osteogenic and adipogenic lineages at passage 3. Osteogenic differentiation of hDPSCs was induced for 3 weeks as previously described.^27^ Cells were seeded at approximately 3 × 10^3^ cells/cm^2^ on culture dishes in osteogenic medium (culture medium supplemented with 5% FCS, 100 μmol/L 2P‐ascorbic acid, 100 nmol/L dexamethasone, 10 mmol/L β‐glycerophosphate) for 4 weeks. Mineralized extracellular matrix deposition was assessed through Alizarin Red staining.

Adipogenic differentiation was performed as previously described.^28^ hDPSCs were seeded on 24‐well plates at a cell density of 2 × 10^4^cells/cm^2^. Subconfluent cultures were incubated in the adipogenic medium (culture medium containing 0.5 mmol/L isobutyl‐methylxanthine, 1 μmol/L dexamethasone, 10 μmol/L insulin, 200 μmol/L indomethacin, 50 mg/mL gentamicin) for 3 weeks. Medium was changed every 3 days. Afterwards, cells were evaluated for the formation of lipid droplets by means of oil red O staining. Undifferentiated cells were used as negative control.

### 2.3 Myogenic differentiation of hDPSCs

In order to evaluate the myogenic differentiation potential of hDPSCs, 4000 cells/cm^2^ were seeded in expansion medium consisting in DMEM‐High Glucose (DMEM‐HG) plus 10% FBS, 2 mmol/L L‐glutamine, 100 U/mL penicillin and 100 μg/mL streptomycin. After reaching 70% confluency, cells were preliminarily treated with the demethylating agent 5‐aza‐2′‐deoxycytidine (5‐Aza; 10 μmol/L) for 24 hours in DMEM low glucose (DMEM‐LG), plus 10% horse serum, 0.5% chicken serum, 2 mmol/L L‐glutamine, 100 U/mL penicillin and 100 μg/mL streptomycin. After 24 hours of demethylating treatment, hDPSCs were processed for evaluation of early myogenic transcription factors (Pax7 and myogenin) by real‐time PCR and Western Blot analysis. Besides hDPSCs were maintained for in myogenic medium (DMEM‐LG, plus 5% horse serum, 0.5% chicken serum, 2 mmol/L L‐glutamine, 100 U/mL penicillin, 100 μg/mL streptomycin and 10 nmol/L insulin) for 3 weeks.^28^ At the end of the differentiation time, the achievement of myogenic commitment was assessed by confocal immunofluorescence and Western Blot analysis of late markers myosin heavy chain and desmin (Sigma‐Aldrich), as described below. Differentiated C2C12 cells were used as positive controls.

### 2.4 Real‐time PCR analysis

Human DPSC cells were homogenized, and total RNA was extracted and purified using the PureLink RNA columns (Thermo Fisher Scientific). cDNA synthesis was performed by using Maxima First Strand cDNA Synthesis Kit with DNase I treatment (Thermo Fisher Scientific). Quantitative real‐time PCRs were performed using SYBR Green Master mix (Bio‐Rad) on CFX Connect Real‐time PCR instrument (Bio‐Rad), with the following oligonucleotides: h RPLP0 (F: TACACCTTCCCACTTGCTGA, R: CCATATCCTCGTCCGACTCC); h Pax7 (GAGGATGAAGCGGACAAGAA, R: TCAGTGGGAGGTCAGGTT); h Myogenin (F: GGTGCCCAGCGAATGC R: TGATGCTGTCCACGATGGA). Relative quantification was calculated from the ratio between the cycle number (Ct) at which the signal crossed a threshold set within the logarithmic phase of the given gene and that of the reference hRPLP0. Mean values of the duplicate results of three independent experiments for each sample were used as individual data for 2‐ΔΔCt statistical analysis.

### 2.5 Western Blot analysis

The myogenic commitment of hDPSCs in vitro was also investigated by Western Blot analysis. Whole cell lysates were obtained as previously described.^25^ Briefly, 30 µg of protein extract per sample was quantified by a Bradford Protein Assay (Bio‐Rad), underwent SDS‐polyacrylamide gel electrophoresis and was then transferred to PVDF membranes. The following antibodies were used: mouse anti‐Pax7 (DSHB, Hybridoma Bank), mouse anti‐myogenin and rabbit anti‐desmin (Sigma‐Aldrich), diluted 1:1000 in Tris‐buffered saline Tween 20 plus 2% BSA and 3% non‐fat milk. Membranes were then incubated for 30 minutes at room temperature with HRP‐conjugated anti‐mouse and anti‐rabbit secondary antibodies, diluted 1:3000. Membranes were then visualized using ECL (enhanced chemiluminescence, Amersham, United Kingdom). Anti‐actin antibody was used as control of protein loading.

Densitometry was performed by Fiji ImageJ software. An equal area was selected inside each band, and the mean of grey levels (in a 0‐256 scale) was calculated. Data were then normalized to values of background and of control actin band.^26^

### 2.6 Induction of urinary incontinence in animal model after pudendal nerve transection

All experimental protocols were reviewed by the Local Animal Ethics Committee (OPBA), University of Modena and Reggio Emilia, Italy, and approved by Ministero della Salute (authorization number 556/2017‐PR released on 7/6/2017). For the study, 12 weeks old female rats weighing 250/300 g (Charles River laboratories, Calco, Lecco, Italy) were used and housed under standard condition. Prior to the surgery procedures, rats were anesthetized by ketamine hydrochloride (Ketavet 100®; Farmaceutici Gellini) plus xylazine hydrochloride (Rompun®; Bayer AG) by intraperitoneal injection and generation of incompetent urethral sphincter model was carried out according to Kim et al^10^ using a bilateral pudendal nerve transection technique. Briefly, a lower midline abdominal incision was made, and the bladder and urethra were exposed. The pudendal nerve on each side was identified and transected with microsurgical scissors under stereomicroscopic magnification. Laparotomy was closed in layers with absorbable 4 to 0 vicryl sutures.

### 2.7 hDPSCs injection

One week after generation of SUI, animals were anesthetized, and the bladder and the urethra were exposed by a lower abdominal incision. Approximately 1 x 10^6^ hDPSCs preconditioned with 5‐AZA for 24 hours, were resuspended in 10 μL of PBS 1X and injected bilaterally in the external sphincter of each animal (5 x 10^5^ cells for each side, at the three and nine o'clock area), by using a 50 μl Hamilton microsyringe pump connected to a needle (170 μm OD; World Precision Instruments. For the study, a total of 24 female rats were used as follows: (a) Control sham‐operated rats (Ctrl; n = 8); (b) Pudendal nerve transection rats without cell injection (cell‐; n = 8); (c) Pudendal nerve transection rats with hDPSCs injection (cell+; n = 8). Animals were sacrificed 4 weeks after cell injection.

### 2.8 Measurement of leak point pressure (LPP)

Four weeks after the injection of hDPSCs, urodynamic functional test was performed to measure the leak point pressure (LPP) in order to evaluate the functional recovery of urethral sphincter in the three experimental groups. As previously described by Kim et al, before measuring, rats were anesthetized, and the spinal cord was transected at the T9‐T10 level to abrogate reflex bladder activity in response to increasing intravesical pressure although without interfering with the spinal continence reflexes of the bladder neck and urethra.^10^ Under general anaesthesia, the bladder was exposed by a midline incision. A suprapubic PE‐50 bladder catheter solidarized to the bladder wall was placed and sutured with prolene 4‐0. The abdominal wall and the skin were then sutured in two layers. The suprapubic catheter was then connected by means of a three‐way valve, either to a pressure transducer (P300 type, Grass Instruments) or to a syringe pump (100 type, KD Scientific). The transducer was then linked to an amplifier (MT 9500 type, Astro‐Med, Inc) and a computer to record pressure data. Subsequently, rats were fixed to a support and placed in vertical position.

After a 30 minutes period of accommodation to the filling of 0.3 cc (which represents half of the bladder capacity in a rat weighing 200 g), the bladder was filled with physiological solution (at a speed of 5 cc/h) in order to simulate the bladder filling and the following increased pressure. Bladder pressure was increased in steps in 1‐ to 3‐cm H_2_O steps from 0 cm H_2_O upwards until visual identification of the loss of urine. The pressure recorded at the point of loss was taken as LPP. Subsequently, bladder pressure was reduced until ceasing of urine leakage. The average of three consecutive LPP records was taken as a reference measurement for each animal. Values were expressed as mean ± standard deviation.

### 2.9 Immunofluorescence analysis of stemness markers and myogenic differentiation in vitro

Immunofluorescence analyses were carried out on hDPSC to confirm the immune selection against c‐Kit and STRO‐1 and to evaluate the myogenic commitment after in vitro induction. Cells were fixed with 4% paraformaldehyde in pH 7.4 phosphate buffer saline (PBS) for 20 minutes and washed in PBS. Where needed, membrane permeabilization was carried out by incubating with Triton‐X100 0.1% in PBS for 5 minutes. After rinsing with PBS, samples were blocked with 3% BSA in PBS for 30 minutes at room temperature and then incubated with the primary antibodies mouse IgM anti‐STRO‐1, rabbit anti‐c‐Kit (Santa Cruz Biotechnology), rabbit anti‐myosin heavy chain and rabbit anti‐desmin (Sigma‐Aldrich), diluted 1:50 in PBS containing 3% BSA, for 1 hour at room temperature. After washing in PBS containing 3% BSA, the samples were incubated for 1 hour at room temperature with the secondary antibodies diluted 1:200 in PBS containing 3% BSA (donkey anti‐mouse Alexa546, goat anti‐rabbit Alexa488; Life Technologies). After washings with PBS, cells nuclei were stained with 1 µg/mL DAPI in PBS for 3 minutes; then, samples were mounted with anti‐fading medium (FluoroMount, Sigma‐Aldrich). Samples not incubated with the primary antibody were used as negative controls.^26^ As formerly described, the multi‐labelling immunofluorescence experiments were carried out avoiding cross‐reactions between primary and secondary antibodies. Samples were observed by a Nikon A1 confocal laser scanning microscope. The confocal serial sections were processed with ImageJ software to obtain three‐dimensional projections, and image rendering was performed using Adobe Photoshop Software.^25^

### 2.10 Histological, morphometrical and immunohistochemical analyses in vivo

Four weeks after hDPSCs injection, the animals were sacrificed, with bladder and urethra being harvested from each rat; then, tissues were fixed in 4% paraformaldehyde in PBS, rinsed with PBS and dehydrated with graded ethanol, cleared and embedded in paraffin. Five micrometer thick serial cross sections of the specimens from each experimental group were obtained. Routine haematoxylin/eosin staining was performed, in order to analyse the morphological details of the tissues. The extent of fibrosis in the urethral sphincter area was determined by using Masson's trichrome staining, according to the manufacturer's instructions (Masson's Trichrome stain kit; Sigma‐Aldrich). Images of the histological samples were obtained with a Nikon Labophot‐2 optical microscope equipped with a DS‐5Mc CCD colour camera. Morphometrical analyses were performed by ImageJ software on three slides per animal per experimental group, 5 weeks after pudendal nerve transection. In particular, the thickness of the striated sphincter was measured to evaluate whether muscular atrophy reversion occurred; the percentage of fibrosis was calculated with respect to the total sphincter area. Vascularization of the injected sphincters was measured by counting vasa—identified through morphological criteria and after immunolabeling against von Willebrand factor—within the striated sphincter. Values were reported as mean ± standard deviation (SD). Engraftment of hDPSCs in the injected sphincters was investigated by immunofluorescence and immunohistochemical analyses, as described above, and the following primary antibodies were used: mouse anti‐human mitochondria (hMit, 1:100; Millipore), rabbit anti‐myosin heavy chain (myo, 1:100; Sigma‐Aldrich), rabbit anti‐von Willebrand factor (vWf, 1:100; Millipore), mouse anti‐neurofilament heavy (NFH; 1:100, Millipore) and mouse anti‐proliferating cell nuclear antigen (PCNA, 1:100; Millipore) antibodies. For immunofluorescence analyses, the following secondary antibodies were used: goat anti‐rabbit Alexa488, goat anti‐mouse Alexa488, goat anti‐rabbit 546 (Life Technologies). Immunohistochemistry with anti‐hMit revealed by an anti‐mouse HRP‐labelled secondary antibody (Thermo Fisher) was performed in order to further confirm the labelling of vasa walls. HRP was revealed by a DAB based kit (Sigma‐Aldrich).

### 2.11 Statistical analysis

All the experimental procedures were performed in triplicate. Values were expressed as mean ± standard deviation (SD). Differences between experimental samples were analysed by ANOVA followed by Newman‐Keuls post hoc tests. Differences between two groups were analysed by Student *t* test (GraphPad Prism Software version 5 Inc). In any case, significance was set at *P* < .05.

## 3 RESULTS

### 3.1 Immune selection of hDPSCs and stemness evaluation

**Figure 1:**
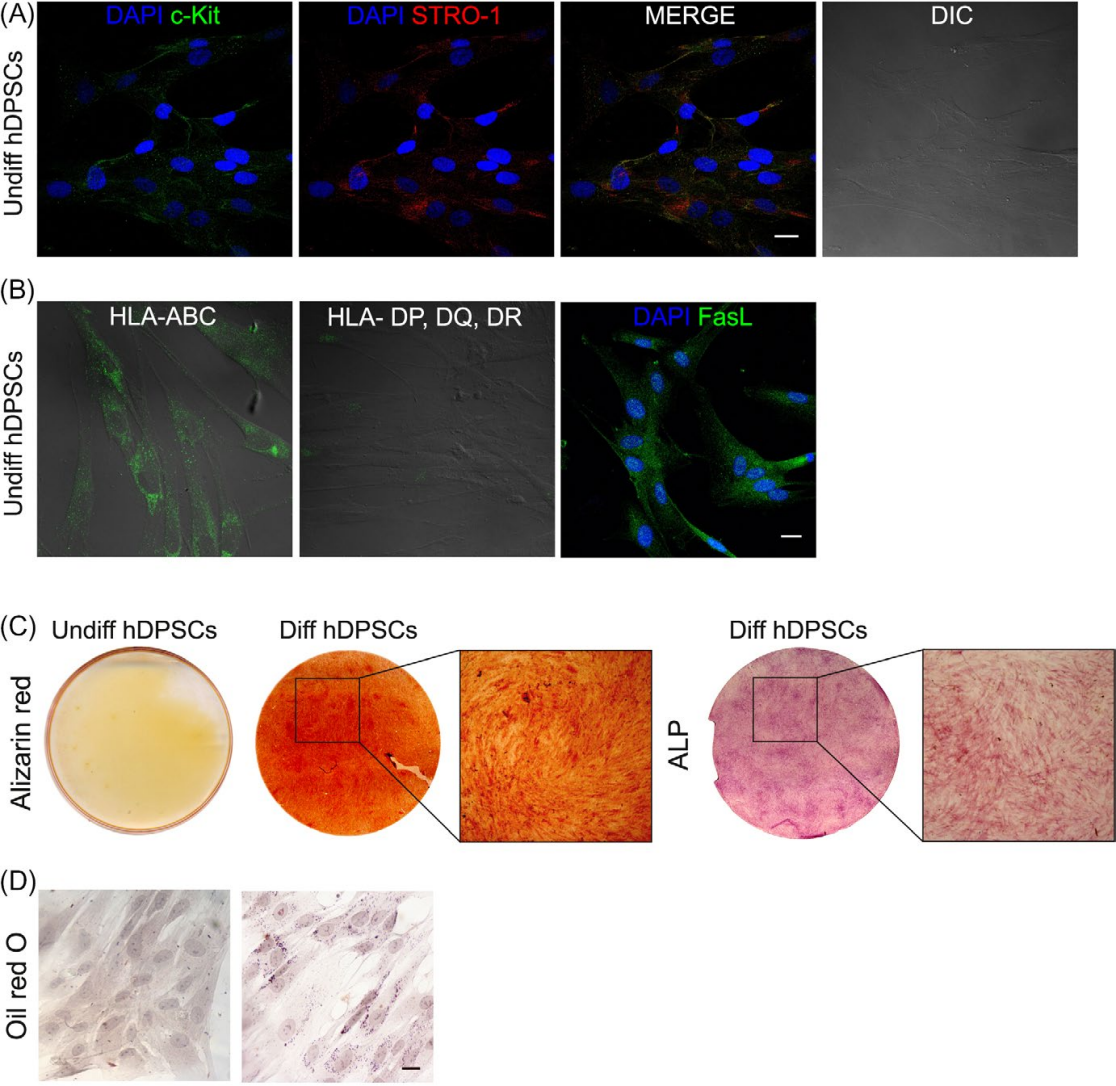
Immune selection and stemness evaluation. A, immunofluorescence analysis of hDPSCs after MACS sorting against c‐Kit and STRO‐1. Nuclei were counterstained with DAPI, and cellular morphology was shown by DIC. B, Immunofluorescence evaluation of HLA‐ABC, HLA‐DP, DQ, DR and FasL. Nuclei were counterstained with DAPI, and cellular morphology was shown by DIC. C, Evaluation of osteogenic commitment of hDPSCs through Alizarin red staining and ALP assay. Undifferentiated cells were used as control. D, Oil red O staining of undifferentiated hDPSCs and hDPSCs after adipogenic induction. Scale bar: 10 μm

Following the double immune‐magnetic cell sorting, hDPSCs were stained for c‐Kit and STRO‐1 surface markers. Almost all the cells showed a fibroblast‐like morphology and labelled positive for both the stemness markers (Figure 1A). These data demonstrate that a homogeneous stem cell population was obtained, starting from the whole hDPSCs population. Moreover, selected stem cells show only the expression of Histocompatibility complex of class 1 (HLA‐ABC) and are negative for the expression of Histocompatibility complex of class 2 (HLA‐DP,DQ,DR). The positive staining for FasL in association with the HLA profiles reveals that hDPSCs selected for c‐Kit and STRO‐1 own the typical mesenchymal stemness properties exerting low immunogenicity and immunomodulatory properties (Figure 1B). After 3 weeks of osteogenic induction, deposition of mineralized extracellular matrix was clearly detected through Alizarin Red staining in differentiated hDPSCs. Osteogenic commitment was also confirmed by Alkaline Phosphatase assay (Figure 1C). Oil red O staining demonstrated that adipogenic differentiation was reached after 3 weeks of culture with specific induction medium, by microscopic observation of lipid droplets formation in committed hDPSCs (Figure 1D). Undifferentiated hDPSCs were used as control.

### 3.2 Myogenic differentiation in vitro

**Figure 2:**
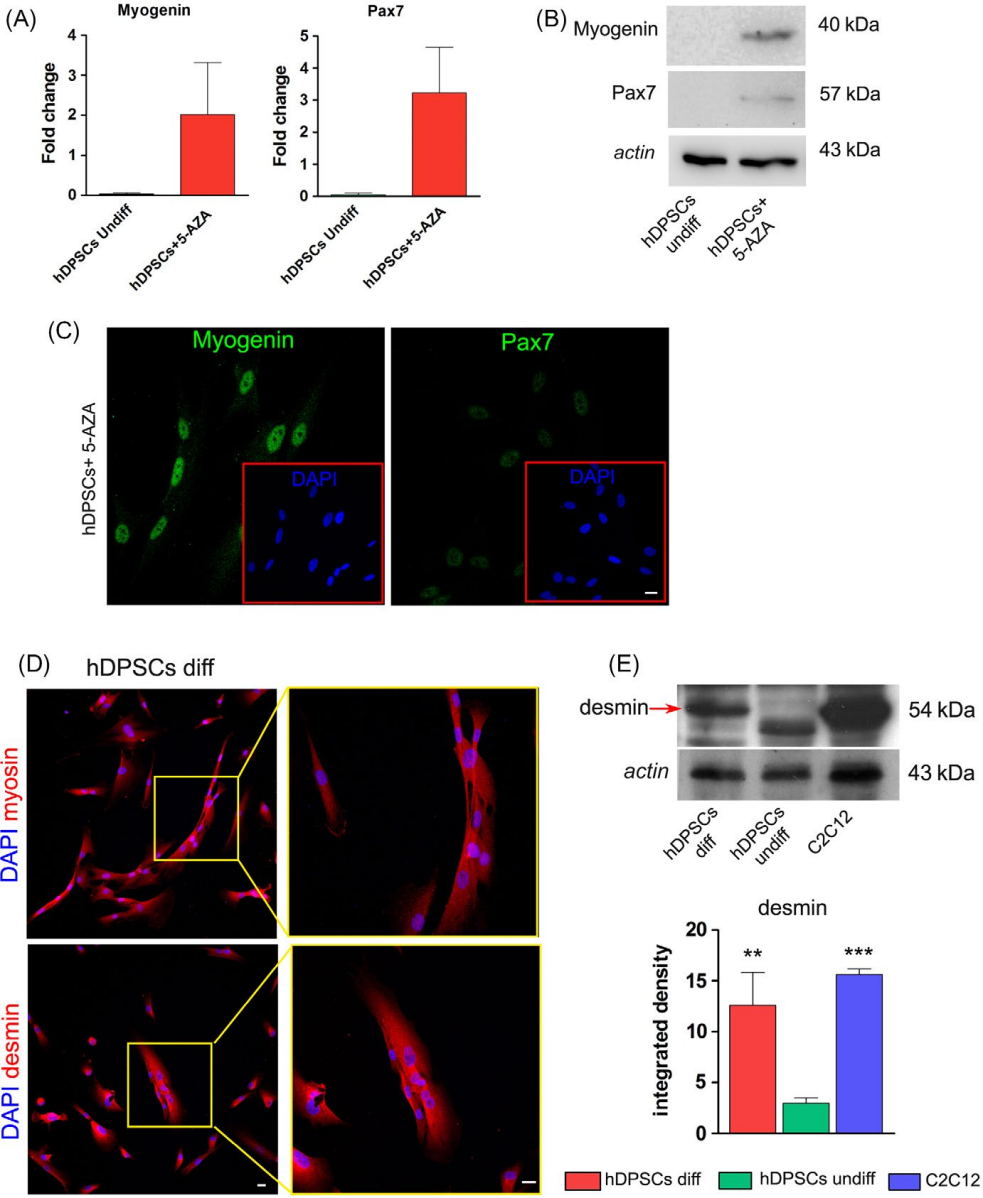
Myogenic commitment and differentiation of hDPSCs. A, Real‐time PCR analysis showing fold increase of mRNA levels of myogenin and Pax7 after 24 h treatment with 5‐Aza. Data represent mean ± SD of fold change obtained from three independent experiments. B, Western blot analysis of myogenin and Pax7 in undifferentiated and 5‐Aza treated hDPSCs. C, Immunofluorescence analysis of myogenin and Pax7 in 5‐Aza treated hDPSCs. D, Immunofluorescence analysis of myosin and desmin in hDPSCs after 3 wk of differentiation. Higher magnification reported on the right shows the presence of multinucleated syncitia. E, The expression of desmin was evaluated by Western Blot analysis after 3 wk of differentiation. The bar graph represents the mean ± SD of integrated density of desmin. ANOVA test followed by Newman‐Keuls post hoc test; **P < .01, ***P < .001 vs undifferentiated hDPSCs (n = 3)

When hDPSCs were cultured in myogenic medium, following 24 hours of demethylation treatment with 10 µmol/L 5‐Aza, a change in cell morphology was observed, with hDPSCs showing an elongated spindle shape (data not shown). The expression of Pax7 and myogenin, which are implicated in the specification of cells that might enter the myogenic program, was evaluated by real‐time PCR and Western Blot analyses as shown in Figure 2A,B. Data demonstrate that, after 24h of preconditioned culture with 5‐Aza, hDPSCs showed a fold increase in mRNA level of Pax7 and myogenin with respect to undifferentiated hDPSCs (Figure 2A). Moreover, Western blot analysis revealed that the demethylating treatment induced the expression of myogenic‐related markers, Pax7 and myogenin, when compared to untreated hDPSCs (Figure 2B). These data were confirmed by immunofluorescence analysis (Figure 2C). After 3 weeks of culture with myogenic medium, hDPSCs showed the formation of multinucleated syncitia expressing the typical late myogenic markers myosin and desmin (Figure 2D). Western blot analysis of desmin confirmed that hDPSCs committed towards myogenic lineage, showing a statistically significant increase of desmin expression in comparison with undifferentiated hDPSCs (**P < .01) (Figure 2E).

### 3.3 Histological and histomorphometric analyses

**Figure 3:**
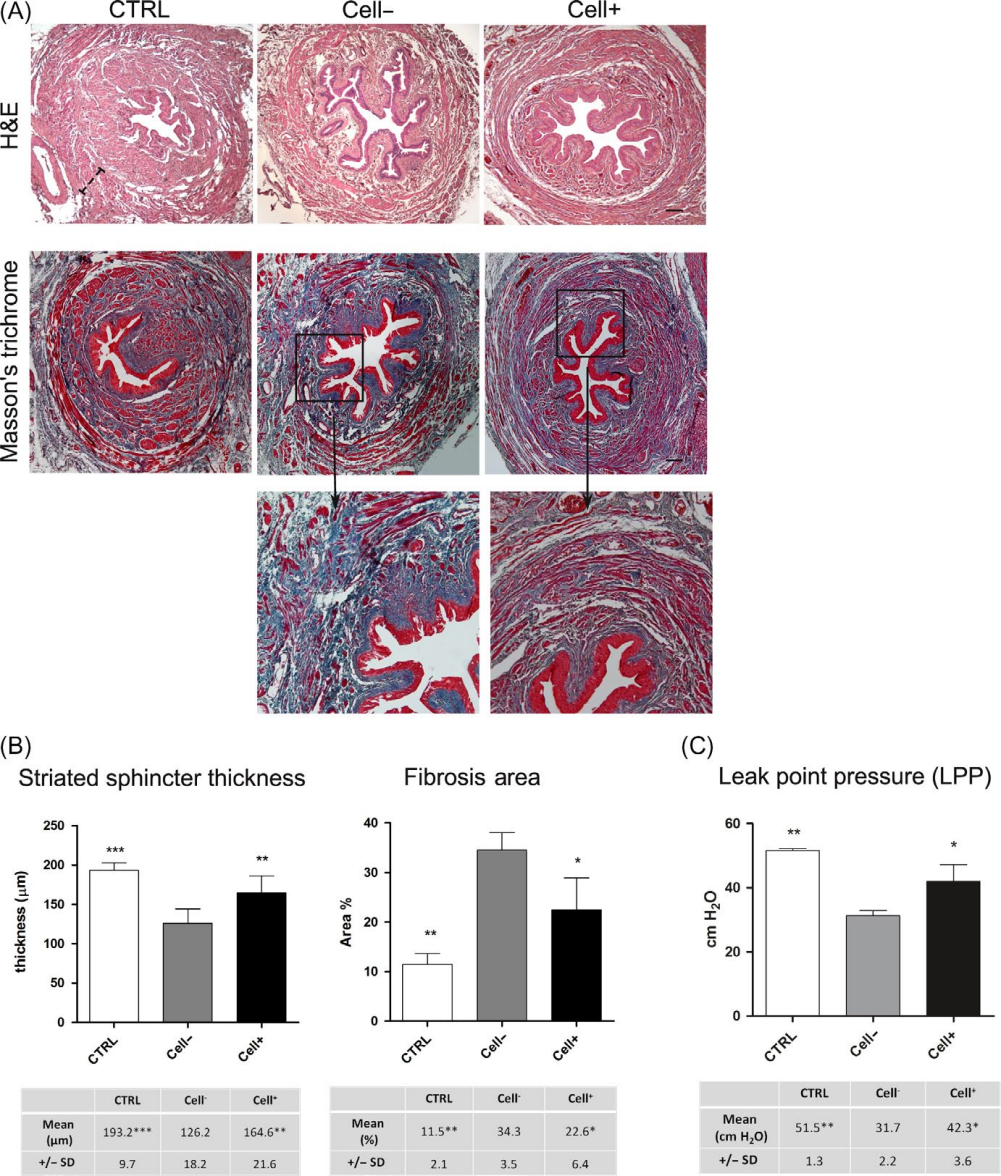
Histological and histomorphometric analyses. A, Histological analysis by H&E staining of urethral sphincter performed in all experimental groups. Masson's trichrome staining showing fibrosis distribution through the whole urethral sphincter section. Scale bar: 100 μm. B, Histograms represent histomorphometric evaluations of striated sphincter thickness and the percentage of fibrosis area in the three experimental groups. C, Histograms report the LPP obtained by urodynamic evaluation in each experimental group. In any case, values were expressed as mean ± SD; *P < .05, **P < .01, ***P < .001 vs Cell‐ group. ANOVA followed by Newman‐Keuls post hoc test

In order to evaluate the urethral sphincter regeneration, serial sections from urethra specimens harvested 5 weeks after pudendal nerve transection were stained by H&E and Masson's trichrome. Histological analysis of H&E stain showed relevant differences in the arrangement of striated fibres of external urethral sphincter among the three experimental groups, with Cell + group exhibiting a histological organization resembling the control group (Figure 3A). Moreover, in the experimental group Cell‐, H&E staining showed the presence of fibrotic tissue spread through the entire tissue section. These data were confirmed by Masson's trichrome staining. In fact, histological analysis revealed that Cell‐ group was characterized by clear fibrosis spread through the striated sphincter area, which was scarcely recovered. On the other hand, Cell + group showed an appreciable reduction of fibrosis in parallel with an ongoing rearrangement of the striated sphincter area, as shown by magnification images in Figure 3A. Based on these observation, histomorphometric analyses were carried out in order to measure the striated sphincter thickness and the fibrosis area. As reported in histograms, the experimental group Cell + showed statistically significant higher thickness of striated sphincter when compared to Cell‐ group (**P < .01 vs Cell‐). At the same time, the mean percentage of fibrosis area was statistically significant lower in Cell + group (*P < .05 vs Cell‐) (Figure 3B). As reported in Figure 3C, urodynamic evaluation demonstrated that LPP mean value was statistically significant higher in Cell + group with respect to Cell‐ group (*P < .05 vs Cell‐), whereas no significant differences were detected between Cell + group and control group, thus demonstrating that urethral sphincter continence was restored following hDPSCs injection.

### 3.4 Engraftment of hDPSCs in striated urethral sphincter and promotion of vascularization

**Figure 4:**
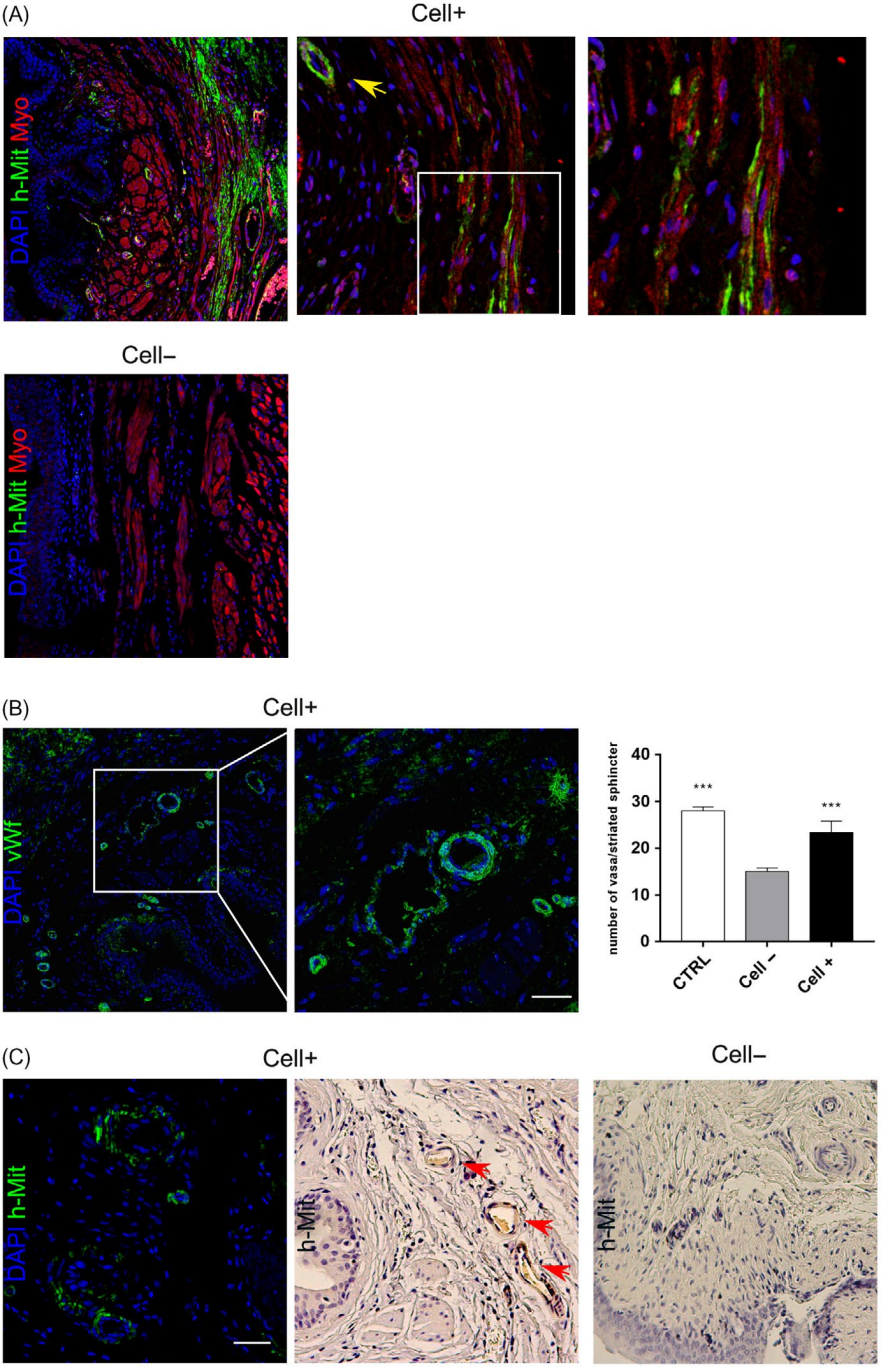
Histointegration of hDPSCs. A, Transverse sections of urethral sphincters were labelled against human mitochondria (green) and myosin (red), in order to evaluate the localization of human stem cells in Cell + tissues. Yellow arrowhead indicating the presence of hMit + cells surrounding vessels like structures. Magnification of the inset (white square) highlights muscle fibres contemporarily labelled against human mitochondria and myosin. Nuclei were counterstained with DAPI. B, Immunofluorescence analysis performed on Cell + transverse sections against von Willebrand factor. On the right, histograms represent the number of vasa in striated sphincter. Values were expressed as mean ± SD; ***P < .001 vs Cell‐ group. ANOVA followed by Newman‐Keuls post hoc test. C, Immunofluorescence and immunohistochemistry analyses of hMit performed in Cell + and Cell‐ groups. Positive staining reveals the signals localization in structures morphologically resembling vessels (red arrowheads). Scale bar: 100 μm

Five weeks after the bilateral transection of pudendal nerve, immunofluorescence analyses were carried out on serial cross sections of urethral sphincters from each experimental group, in order to evaluate the presence of hDPSCs in the injected areas and their contribution to continence recovery. As shown in Figure 4A, immunofluorescent staining with anti‐myosin antibody allowed to identify the muscular component. The hMit labelling identified the presence of hDPSCs localized in the peripheral area of the tissue, specifically in the striated muscle fibres of urethral sphincter. Moreover, hMit^+^ cells were also detected in structures morphologically resembling vasa (Figure 4A, yellow arrowhead). Cell‐ group was used as negative control.

In order to better evaluate the vascularization in hDPSCs‐injected groups, immunofluorescence analyses were carried out using anti‐von Willebrand factor antibody (Figure 4B). The number of vasa was measured in the striated sphincter from each experimental group, and as reported in histograms, Cell + group exhibited a statistically significant higher number of vasa when compared to Cell‐ group (***P < .001). Data demonstrate that the number of vasa observed in Cell + group was close, although slightly lower, to control group.

Immunofluorescent and immunohistochemical labelling against hMit show areas with hMit^+^ cells arranging to form structures resembling vessels in the urethral sphincter (Figure 4C).

### 3.5 Human DPSCs home to the injured nerve

Stress urinary incontinence animal model was created by transecting the pudendal nerve, which subsequently produced the muscle atrophy mimicking the pathophysiology of SUI.

**Figure 5:**
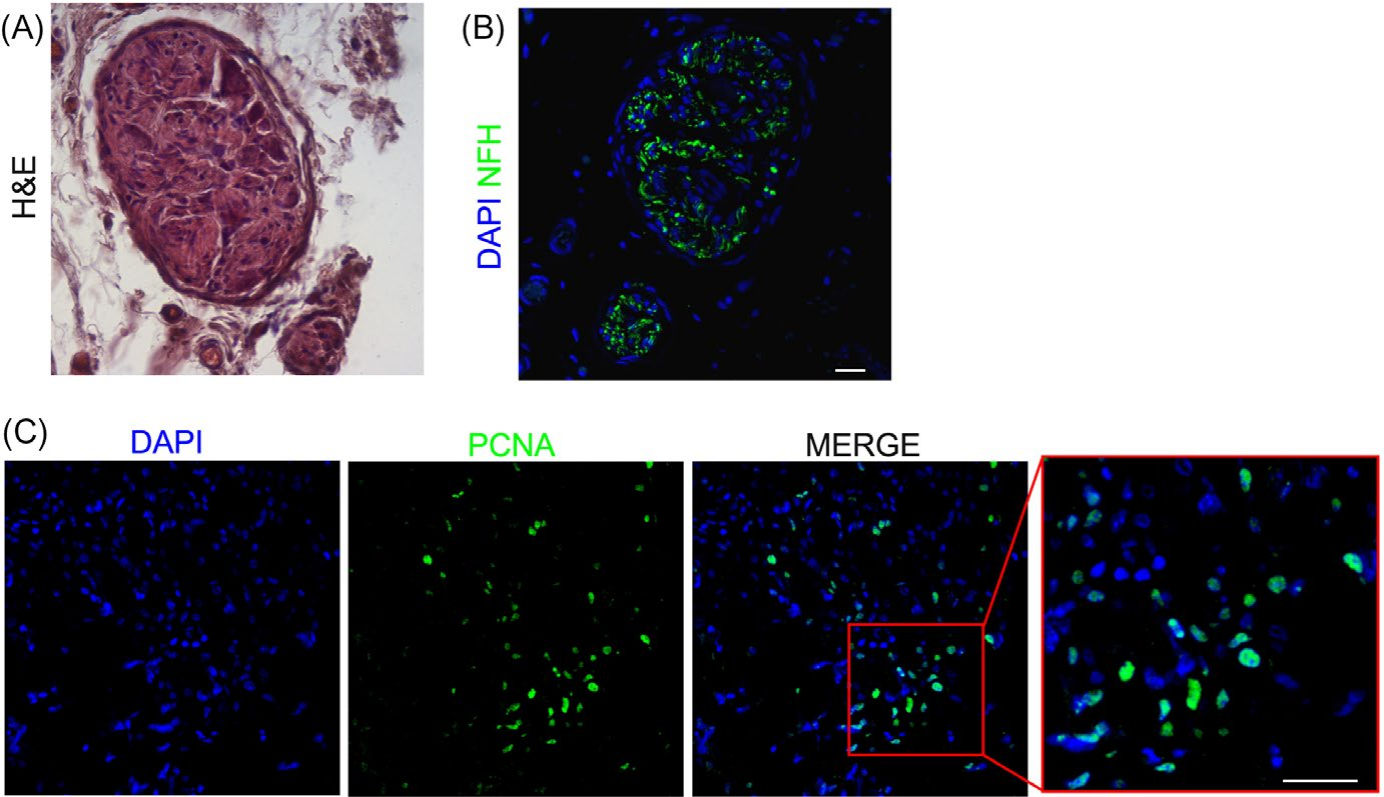
Integration of hDPSCs into the injured nerve. A, H&E staining of a cross section highlights the pudendal nerve. B, immunofluorescence staining against neurofilament heavy (NFH, green) confirms the arrangement of pudendal nerve fibres. C, Immunofluorescence labelling against human PCNA (green) shows the presence of hDPSCs within the regenerating pudendal nerve. Nuclei were counterstained with DAPI. Scale bar: 50 μm

The histological analysis of cross sections obtained from Cell + group—stained by H&E—allowed to recognize the anatomical localization of pudendal nerve and, as reported in Figure 5A, only a limited fibrosis was present. Particularly, as reported in Figure 5B, confocal immunofluorescence analysis confirmed the presence of partial and discontinuous alignment of regenerating fibres as shown by the positive staining for Neurofilament heavy. Moreover, in transverse sections the presence of human PCNA^+^ cells was observed. These data suggest the possible role of hDPSCs in homing to the injured nerve and their contribution in promoting nerve regeneration (Figure 5C).

## 4 DISCUSSION

Urinary incontinence (UI), occurring as an involuntary leakage of urine, originates from the impairment of striated muscle function in the urethra, due to stretch, torn or crush of pudendal nerve. In women, the most important factors that may trigger the onset of stress urinary incontinence are pregnancies, obesity and menopause, while in men SUI is mainly associated with surgical iatrogenic lesions (ie after radical prostatectomy or endoscopic resection of prostate). These factors are the basis of tissue damage, both muscular and nervous, which leads to alteration of the sphincter function. The evaluation of the presence and degree of incontinence must be done through an accurate diagnostic procedure. It may be necessary to collect the patient's medical history, perform a thorough physical examination, use specific and validated questionnaires differentiated for male and female,^29^, ^30^, ^31^ obtain completed voiding diaries,^32^ exclude urinary infections,^33^ evaluate post‐void residual volume,^34^ perform urodynamic testing,^35^ pad testing^36^ and imaging.^37^

In clinical practice, urinary incontinence is often associated with multiple comorbidity conditions. For this reason, non‐surgical therapies are generally used first.

These may consist in correction of underlying diseases associated with incontinence^38^ (ie diabetes, neurological and cognitive diseases, sleep disorders), treatment of constipation,^39^ lifestyle interventions^40^, ^41^, ^42^ (ie weight loss, cessation of smoking, increase in physical activity), behavioural and physical therapies^43^ (ie prompted voiding in patients with behavioural disorders, bladder training, pelvic floor muscle training, electrical stimulation).

In the category of non‐surgical treatments, there are also pharmacological managements. Pharmacological treatments are more effective in the treatment of urge incontinence; however, in selected cases of SUI, duloxetine, oestrogens and desmopressin can find their application.^44^, ^45^, ^46^

In cases, where the SUI cannot be resolved with the treatments listed above, surgery remains the therapeutic option. The surgeons’ experience performing these procedures might influence the results.^47^ The therapeutic options range from the application of mid‐urethral slings to the open or laparoscopic or robotic colposuspension,^48^ to the injection of bulking agents^49^ and the placement of artificial sphincters.^50^ Furthermore, as a result of radical prostatectomy, the repair with fibrous tissue is frequent, which could cause these devices to be difficult to use and ineffective.

It is important to underline how the slings in women are tension‐free, while in men these can result in decubitus at urethral level, with the risk of erosions. Consequently, conventional therapies including pharmacology and surgery approaches offer appreciable results although they do not provide a complete resolution of the disease. Indeed, despite the high number of available procedures, not all patients can benefit from surgery, because the risks related to the procedure (associated with patients’ comorbidities) may outweigh the benefits of this therapy.^51^ For several years, cell therapy based regenerative medicine has been investigated as a valid alternative to the normal therapeutic options in order to reach the resolution of the pathophysiology by regenerating the injured tissue.^52^

Currently, the use of stem cells might represent an appealing alternative to restore urethral sphincter continence for the treatment of SUI. Up to now, many different stem cells sources have been investigated for their potential application to SUI.^10^, ^11^, ^12^, ^13^, ^14^, ^15^, ^16^, ^17^, ^21^, ^23^, ^53^, ^54^, ^55^, ^56^ Previous findings from our group have extensively demonstrated the myogenic potential of hDPSCs and their ability to ameliorate the histopathology of dystrophic skeletal muscle in SCID/mdx model.^24^ Based on these results, the aim of our study was to apply hDPSCs in the regeneration of striated muscle atrophy induced by a primary nerve injury. To this regard, the model of pudendal nerve transection, which triggers the muscle atrophy resulting in urinary incontinence, represents a suitable experimental approach to study the potential application of hDPSCs in the treatment of SUI.

Data demonstrate that after 3 weeks of myogenic induction in vitro sorted hDPSCs were able to differentiate towards myogenic commitment by forming multinucleated syncytia and by expressing late myogenic markers. Noteworthy, the use of demethylating agent 5‐Aza for 24 hours was able to induce the expression of the early markers such as Pax7 and myogenin. This approach was used as pre‐conditioning method prior to stem cells injection in rat urethral sphincter. As a matter of fact, immunofluorescence analysis of Cell + group demonstrated that the majority of human stem cells were entrapped in striated urethral sphincter. Interestingly, we noticed that after treatment a considerable reduction of fibrosis occurred in parallel with the recovery of muscle atrophy. This process might be sustained by the contribution of hDPSCs either in forming new syncytia or in surrounding vessels. These events are known to be primary in supporting the reduction of fibrosis process.^57^ Indeed, vascularization promotion is a well‐documented ability characterizing hDPSCs^24^, ^58^, ^59^ which does not relate to tumorigenesis, being instead important for regeneration. As formerly demonstrated by Sun et al,^60^ vascularization proved to be a favourable condition in supporting the urethral regeneration. The regeneration of muscular component only would provide a transient/temporary recovery of the urinary continence. Interestingly, our findings revealed the presence of hDPSCs not only in the injection tissue but also in transected injured nerve fibres, thus confirming previous evidence from our group regarding the capability of hDPSCs to sustain nerve regeneration. It is important to highlight that muscle atrophy is a secondary condition of pudendal nerve injury^10^; therefore, a cell therapy restoring either muscle trophism or supporting nerve repair would represent a suitable tool for the treatment of SUI. In consideration of our results and according to previous findings that demonstrated the low immunogenic human dental pulp stem cells and their ability to modulate the immune system in vitro by direct cell‐cell contact or by secreting different soluble factors under allogeneic condition, the next step might be a deeper investigation of the immunomodulatory pathway in vivo.

## CONFLICT OF INTERESTS

The authors declare that no conflict of interests exists.

## Data Availability

The data that support the findings of this study are available from the corresponding author upon reasonable request.
